# Bis(2,2′-bi-1*H*-imidazole)­copper(II) bis­(1,1,3,3-tetra­cyano-2-eth­oxy­propenide)

**DOI:** 10.1107/S1600536810029752

**Published:** 2010-07-31

**Authors:** Bachir Gaamoune, Zouaoui Setifi, Adel Beghidja, Malika El-Ghozzi, Fatima Setifi, Daniel Avignant

**Affiliations:** a Faculté des Sciences, Département de Chimie, Université Ferhat Abbas de Sétif, 19000 Sétif, Algeria; b Unité de Recherche de Chimie de l’Environnement et Moléculaire Structurale, CHEMS, Faculté des Sciences Exactes, Département de Chimie, Université Mentouri Constantine, 25000 Constantine, Algeria; c Laboratoire des Matériaux Inorganiques, UMR CNRS 6002, Université Blaise Pascal, 24 Avenue des Landais, 63177 Aubière, France

## Abstract

In the title compound, [Cu(C_6_H_6_N_4_)_2_](C_9_H_5_N_4_O)_2_, the Cu^2+^ ion (site symmetry 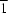) is coordinated by two *N*,*N*′-bidentate 2,2′-biimidazole (H_2_biim) ligands, generating a square-planar CuN_4_ geometry. The dihedral angle between the aromatic rings in the ligand is 0.70 (9)°. In the polynitrile 1,1,3,3-tetra­cyano-2-eth­oxy­propenide (tcnoet) anion, the C—N, C—C and C—O bond lengths indicate extensive electronic delocalization. An alternative description for the metal-ion geometry is an extremely distorted CuN_6_ octa­hedron, with two N-bonded tcnoet anions completing the coordination. In the crystal, the components are linked by N—H⋯N and C—H⋯N inter­actions.

## Related literature

For the structures and properties of related compounds containing polynitrile anions, see: Atmani *et al.* (2008 ▶); Batten & Murray (2003 ▶); Bencini & Mani (1988 ▶); Benmansour *et al.* (2007 ▶); Cancela *et al.* (2001 ▶); Cromer *et al.* (1987 ▶); Jones *et al.* (2006 ▶); Setifi *et al.* (2006 ▶, 2007 ▶); Thétiot *et al.* (2003 ▶); Triki *et al.* (2005 ▶); Yuste *et al.* (2007 ▶). For the synthesis of the H_2_biim and Ktcnoet ligands, see: Bernarducci *et al.* (1983 ▶) and Middleton & Engelhardt (1958 ▶), respecively. 
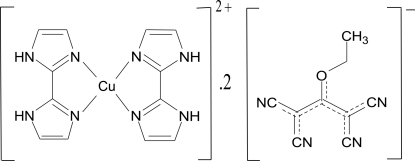


## Experimental

### 

#### Crystal data

[Cu(C_6_H_6_N_4_)_2_](C_9_H_5_N_4_O)_2_
*M* *_r_* = 702.18
Monoclinic, 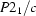

*a* = 8.1001 (8) Å
*b* = 26.1834 (11) Å
*c* = 8.2185 (7) Å
β = 117.086 (11)°
*V* = 1551.9 (2) Å^3^
*Z* = 2
Mo *K*α radiation
μ = 0.76 mm^−1^
*T* = 170 K
0.40 × 0.30 × 0.20 mm

#### Data collection

Oxford Diffraction Xcalibur CCD diffractometer
Absorption correction: multi-scan (*CrysAlis RED*; Oxford Diffraction, 2007 ▶) *T*_min_ = 0.750, *T*_max_ = 0.862
8646 measured reflections
3002 independent reflections
1854 reflections with *I* > 2σ(*I*)
*R*_int_ = 0.037

#### Refinement

*R*[*F* ^2^ > 2σ(*F* ^2^)] = 0.034
*wR*(*F* ^2^) = 0.077
*S* = 0.93
3002 reflections
223 parameters
H-atom parameters constrained
Δρ_max_ = 0.38 e Å^−3^
Δρ_min_ = −0.28 e Å^−3^

### 

Data collection: *CrysAlis CCD* (Oxford Diffraction, 2007 ▶); cell refinement: *CrysAlis RED* (Oxford Diffraction, 2007 ▶); data reduction: *CrysAlis RED*; program(s) used to solve structure: *SIR97* (Altomare *et al.*, 1999 ▶); program(s) used to refine structure: *SHELXL97* (Sheldrick, 2008 ▶); molecular graphics: *ORTEP-3* (Farrugia, 1997 ▶) and *CAMERON* (Watkin *et al.*, 1993 ▶); software used to prepare material for publication: *WinGX* (Farrugia, 1999 ▶) and *PLATON* (Spek, 2009 ▶).

## Supplementary Material

Crystal structure: contains datablocks global, I. DOI: 10.1107/S1600536810029752/hb5555sup1.cif

Structure factors: contains datablocks I. DOI: 10.1107/S1600536810029752/hb5555Isup2.hkl

Additional supplementary materials:  crystallographic information; 3D view; checkCIF report

## Figures and Tables

**Table:** 

Cu1—N1	1.9727 (19)
Cu1—N2	2.0397 (18)
Cu1—N7	2.821 (2)

**Table:** 

N1—Cu1—N2	82.04 (8)

**Table 2:** Hydrogen-bond geometry (Å, °)

*D*—H⋯*A*	*D*—H	H⋯*A*	*D*⋯*A*	*D*—H⋯*A*
N3—H3⋯N9^i^	0.88	2.22	3.011 (3)	149
N4—H4⋯N9^i^	0.88	2.17	2.967 (3)	150
C3—H3*A*⋯N8^ii^	0.95	2.42	3.187 (3)	138

Symmetry codes: (i) 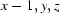; (ii) 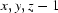.

**Fig. 1.**
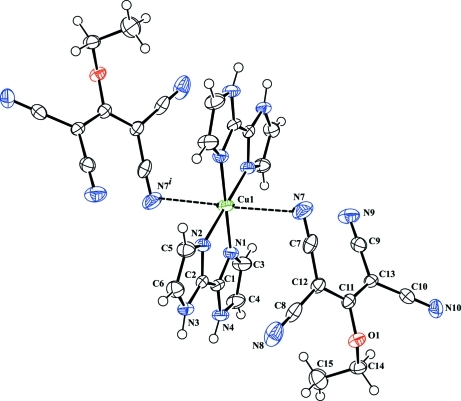
The metal environnement in (I), with displacement ellipsoids drawn at the 50% probability level. Unlabelled atoms are related to labelled atoms by the symmetry operation 1 - x, 1 - y, 1 - z.

**Fig. 2.**
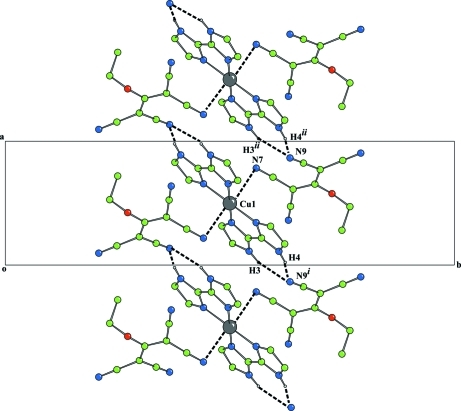
View of the one one-dimensional hydrogen bonded chain on the ab plane. Symmetry codes: i(-1 + x,y,z), ii(1 + x,y,z)

**Fig. 3.**
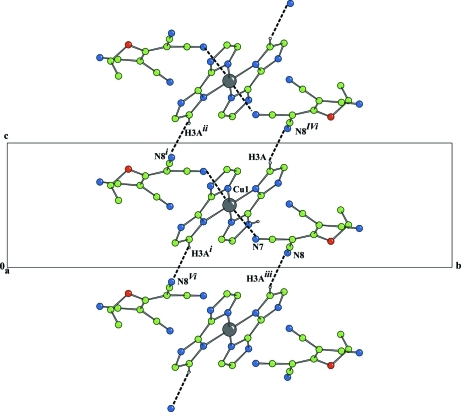
View of the hydrogen bonded maintaining the chains along the c axis. Symmetry codes i(1 - x,1 - y,1 - z), ii(1 - x,1 - y,-z), iii(x,y,1 + z), VIi(x,y,-1 +z), Vi(1 - x,1 - y,2 - z).

## supplementary crystallographic information

### Comment

Polynitrile anions are known to be interesting ligands in coordination chemistry because of their high electronic delocalization and their cyano groups juxtaposed in such a way that they cannot all coordinate to the same metal ion. They adopt different bridging or nonbridging coordination modes wich afford discrete or extended molecular architectures (Atmani *et al.*, 2008; Benmansour *et al.*, 2007; Triki *et al.*, 2005; Thétiot *et al.*, 2003). Following these structural and electronic characteristics, several series of binary systems "polynitrile/*M*(II)" with only polynitrile bridges or ternary systems "polynitrile/co-ligand/*M*(II)" (*M*(II), transition metal ion) involving an additional bridging or chelate co-ligand have been reported. Most of them display one-dimensional, two-dimensional and three-dimensional polymeric assemblies, in which the polynitrile anions act as µ2-, µ3- and/or µ4- bridging ligands and exhibit unusual magnetic properties (Batten & Murray, 2003; Jones *et al.*, 2006; Yuste *et al.*, 2007; Setifi *et al.*, 2006; Setifi *et al.*, 2007).

In our case we have chosen to investigate the ternary system including 2,2'-biimidazole (H_2_bim) selected as co-ligand because it is bifunctional: the imino moieties can be coordinated to a metal ion acting as the first coordination sphere and the amino N—H and C—H groups, as the second coordination sphere, may donate multifold hydrogen bonds to tcnoet anions, extending the structure into a high-dimensional network. In this contribution we report the synthesis and the crystal structure of a new copper(II) compound with neutral 2,2'-biimidazole, Cu(H_2_biim)_2_] (tcnoet)_2_, (I).

The crystal of (I) is built of [Cu(H_2_biim)_2_]^2+^ cations and (tcnoet)^-^ anions interconnected by hydrogen bonds. As shown in Fig. 1, the Cu(II) ion has a square coordination geometry, it locates on a symmetry inversion center and relates four nitrogen atoms of two symmetry-related 2,2'-biimidazole molecules which bind bidentately arranged *trans* to each other in the square plane [Cu1—N1 = 1.973 (2) Å and Cu1—N2 = 2.040 (2) Å] and interacts with two nitrogen atoms belonging to tcnoet ligands occupying the apical coordination sites [Cu1—N7 = 2.821 (3) Å]. Selected interatomic distances and angles are listed in Table 1.

The Cu—N bond distances to H_2_biim and inter-ring C1—C2 bond length in (I) present no unusual features and are consistent with the previous report in [Cu(H_2_biim)_2_]Cl_2_ [Bencini & Mani, 1988], [Cu(Me_4_biim)ONO_2_]Cl [Bernarducci *et al.*, 1983] and [VOCl(H_2_Biim)_2_]Cl [Cancela *et al.*, 2001] complexes. In our case the principal coordination is planar and the Cu atom lies within that plane. Both imidazole rings are planar, with no atoms deviating by more than 0.007 A° from the least-squares plane. The two rings of H_2_biim are nearly coplanar, making an angle of 0.70 (9)°. This value compares well with that found in the mononuclear copper(II) species [Cu(H_2_biim)_2_]^2+^ which are in a strictly planar environment (Bencini & Mani, 1988) and that observed in the free H_2_biim molecule (Cromer *et al.*, 1987), but it is smaller than that found for the mononuclear oxovanadium(IV) species [VOCl(H_2_biim)_2_]Cl (Cancela *et al.*, 2001).

[Cu(H_2_biim)_2_](tcnoet)_2_ units are connected to each other *via* hydrogen bonds N—H···N resulting in a one-dimensional chains as shown in Fig. 2. Furthermore these chains are maintained through van der Waals interactions on the (*ab*) plane and connect each other *via* C—H···N hydrogen bonds into a two-dimensional network (Fig. 3). Interestingly, each tcnoet^-^ anion help to sustain the one-dimensional assembly and at the same time the final two-dimensional array.

In this complex, the three central C atoms (C11, C12 and C13) of the anionic ligand present an *sp*^2^ hybridization as indicated by the sum of the three angles around them (359.98° or 360.0°). Two additional facts support the idea of electron delocalization over the three central C atoms: (i) the six central C—C bond distances (1.389 A° -1.428 A°) are longer than a normal C═C double bond (1.340 A°) and close to those of benzene and (ii) the C11—O1 bond distance 1.352 A° is much shorter than tne normal C—O single bond, suggesting that the two central and the C11—O1 bond present a partial double character.

### Experimental

H_2_biim and Ktcnoet ligands were synthesized with the published procedures respectively (Bernarducci *et al.*, 1983; Middleton *et al.*, 1958). To a methanolic suspension of H_2_biim (0.025 g, 5 ml) was added drowpize a solution of CuCl_2_.2H_2_O (0.032 g, 5 ml) resulting in a green solution. Ktcnoet was dissolved in water (0.084 g, 10 ml) and was added quickly to the former solution. The final solution was filtred and allowed to evaporate for a week, giving green blocks of (I). *X* band EPR spectrum from a polycristalline powdred sample of (I) recorded at room temperature exhibits a well defined axial signal with g_parallel_ = 2.27 > g_perpendicular_ = 2.07 consistent with a Cu(II) monomer.

### Refinement

All H atoms were placed in geometrical positions and refined using a riding model, with C—H distances in the range 0.95–0.99 Å and their displacement parameters were set to *U*_iso_(H) = 1.5*U*_eq_(C) for methyl group and *U*_iso_(H) = 1.2*U*_eq_ (C) for all others, while N—H bond lengths were fixed to 0.88 Å with *U*_iso_(H) = 1.2*U*_eq_(carrier N atom).

### Crystal data

**Table:**

[Cu(C_6_H_6_N_4_)_2_](C_9_H_5_N_4_O)_2_	*F*(000) = 718
*M**_r_* = 702.18	*D*_x_ = 1.503 Mg m^−^^3^
Monoclinic, *P*2_1_/*c*	Mo *K*α radiation, λ = 0.71073 Å
Hall symbol: -P 2ybc	Cell parameters from 3092 reflections
*a* = 8.1001 (8) Å	θ = 2.8–31.5°
*b* = 26.1834 (11) Å	µ = 0.76 mm^−^^1^
*c* = 8.2185 (7) Å	*T* = 170 K
β = 117.086 (11)°	Block, green
*V* = 1551.9 (2) Å^3^	0.40 × 0.30 × 0.20 mm
*Z* = 2	

### Data collection

**Table:**

Oxford Diffraction Xcalibur CCD diffractometer	3002 independent reflections
Radiation source: Enhance (Mo) X-ray Source	1854 reflections with *I* > 2σ(*I*)
graphite	*R*_int_ = 0.037
Detector resolution: 8.3622 pixels mm^-1^	θ_max_ = 26.0°, θ_min_ = 2.8°
ω and φ scans	*h* = −9→9
Absorption correction: multi-scan (*CrysAlis RED*; Oxford Diffraction, 2007)	*k* = −30→32
*T*_min_ = 0.750, *T*_max_ = 0.862	*l* = −10→9
8646 measured reflections	

### Refinement

**Table:**

Refinement on *F*^2^	Primary atom site location: structure-invariant direct methods
Least-squares matrix: full	Secondary atom site location: difference Fourier map
*R*[*F*^2^ > 2σ(*F*^2^)] = 0.034	Hydrogen site location: inferred from neighbouring sites
*wR*(*F*^2^) = 0.077	H-atom parameters constrained
*S* = 0.93	*w* = 1/[σ^2^(*F*_o_^2^) + (0.0371*P*)^2^] where *P* = (*F*_o_^2^ + 2*F*_c_^2^)/3
3002 reflections	(Δ/σ)_max_ < 0.001
223 parameters	Δρ_max_ = 0.38 e Å^−^^3^
0 restraints	Δρ_min_ = −0.28 e Å^−^^3^

### Special details

**Table:**

Geometry. All e.s.d.'s (except the e.s.d. in the dihedral angle between two l.s. planes) are estimated using the full covariance matrix. The cell e.s.d.'s are taken into account individually in the estimation of e.s.d.'s in distances, angles and torsion angles; correlations between e.s.d.'s in cell parameters are only used when they are defined by crystal symmetry. An approximate (isotropic) treatment of cell e.s.d.'s is used for estimating e.s.d.'s involving l.s. planes.
Refinement. Refinement of *F*^2^ against ALL reflections. The weighted *R*-factor *wR* and goodness of fit *S* are based on *F*^2^, conventional *R*-factors *R* are based on *F*, with *F* set to zero for negative *F*^2^. The threshold expression of *F*^2^ > σ(*F*^2^) is used only for calculating *R*-factors(gt) *etc*. and is not relevant to the choice of reflections for refinement. *R*-factors based on *F*^2^ are statistically about twice as large as those based on *F*, and *R*- factors based on ALL data will be even larger.

### Fractional atomic coordinates and isotropic or equivalent isotropic displacement parameters (Å^2^)

**Table:**

	*x*	*y*	*z*	*U*_iso_*/*U*_eq_	
Cu1	0.5000	0.5000	0.5000	0.02739 (14)	
O1	0.5765 (2)	0.72753 (6)	0.7896 (2)	0.0279 (4)	
N1	0.3433 (3)	0.55852 (7)	0.3669 (3)	0.0270 (5)	
N2	0.3432 (3)	0.50383 (7)	0.6359 (2)	0.0250 (4)	
N3	0.1123 (3)	0.54298 (7)	0.6550 (3)	0.0276 (5)	
H3	0.0198	0.5642	0.6311	0.033*	
N4	0.1107 (3)	0.60926 (7)	0.3218 (3)	0.0301 (5)	
H4	0.0189	0.6235	0.3346	0.036*	
N7	0.7829 (4)	0.55883 (8)	0.7720 (3)	0.0473 (7)	
N8	0.3624 (4)	0.63047 (9)	0.8836 (3)	0.0523 (7)	
N9	0.8650 (3)	0.63404 (8)	0.4902 (3)	0.0431 (6)	
N10	0.9040 (3)	0.79164 (8)	0.6867 (3)	0.0388 (6)	
C1	0.2179 (3)	0.57061 (8)	0.4219 (3)	0.0240 (6)	
C2	0.2177 (3)	0.54105 (8)	0.5686 (3)	0.0235 (6)	
C3	0.3131 (4)	0.59086 (9)	0.2255 (4)	0.0362 (7)	
H3A	0.3820	0.5912	0.1579	0.043*	
C4	0.1704 (4)	0.62228 (9)	0.1967 (4)	0.0365 (7)	
H4A	0.1211	0.6484	0.1069	0.044*	
C5	0.3157 (4)	0.48229 (9)	0.7740 (3)	0.0306 (6)	
H5	0.3858	0.4548	0.8490	0.037*	
C6	0.1746 (3)	0.50594 (9)	0.7877 (3)	0.0306 (6)	
H6	0.1278	0.4985	0.8721	0.037*	
C7	0.7098 (4)	0.59593 (10)	0.7744 (3)	0.0312 (7)	
C8	0.4741 (4)	0.63526 (9)	0.8363 (3)	0.0335 (7)	
C9	0.8243 (4)	0.66396 (10)	0.5669 (4)	0.0297 (6)	
C10	0.8449 (3)	0.75144 (10)	0.6727 (3)	0.0286 (6)	
C11	0.6541 (3)	0.68945 (8)	0.7366 (3)	0.0234 (6)	
C12	0.6126 (3)	0.64089 (8)	0.7755 (3)	0.0255 (6)	
C13	0.7714 (3)	0.70132 (8)	0.6596 (3)	0.0241 (6)	
C14	0.4716 (3)	0.76732 (9)	0.6587 (3)	0.0303 (6)	
H14A	0.5344	0.7762	0.5836	0.036*	
H14B	0.4669	0.7984	0.7252	0.036*	
C15	0.2811 (4)	0.74960 (11)	0.5388 (4)	0.0507 (8)	
H15A	0.2125	0.7766	0.4517	0.076*	
H15B	0.2185	0.7413	0.6132	0.076*	
H15C	0.2860	0.7191	0.4720	0.076*	

### Atomic displacement parameters (Å^2^)

**Table:**

	*U*^11^	*U*^22^	*U*^33^	*U*^12^	*U*^13^	*U*^23^
Cu1	0.0314 (3)	0.0214 (2)	0.0359 (3)	0.0069 (2)	0.0209 (2)	0.0044 (2)
O1	0.0385 (11)	0.0222 (8)	0.0283 (9)	0.0026 (8)	0.0200 (9)	0.0005 (8)
N1	0.0270 (13)	0.0232 (11)	0.0362 (13)	0.0033 (10)	0.0191 (11)	0.0019 (10)
N2	0.0249 (12)	0.0226 (10)	0.0291 (11)	0.0045 (10)	0.0137 (9)	0.0026 (10)
N3	0.0281 (13)	0.0241 (11)	0.0352 (12)	0.0040 (10)	0.0183 (11)	−0.0030 (10)
N4	0.0303 (13)	0.0236 (11)	0.0426 (14)	0.0087 (10)	0.0221 (11)	0.0039 (10)
N7	0.0602 (18)	0.0228 (12)	0.0658 (17)	0.0040 (12)	0.0348 (14)	0.0053 (12)
N8	0.078 (2)	0.0439 (15)	0.0591 (17)	−0.0250 (14)	0.0521 (16)	−0.0141 (13)
N9	0.0543 (17)	0.0358 (13)	0.0539 (15)	0.0115 (12)	0.0376 (14)	0.0048 (12)
N10	0.0317 (14)	0.0336 (13)	0.0491 (15)	−0.0085 (12)	0.0165 (12)	0.0003 (12)
C1	0.0268 (16)	0.0156 (12)	0.0318 (15)	0.0018 (11)	0.0154 (13)	−0.0030 (11)
C2	0.0231 (15)	0.0173 (12)	0.0320 (15)	−0.0031 (11)	0.0141 (12)	−0.0067 (11)
C3	0.0447 (19)	0.0298 (15)	0.0478 (18)	0.0104 (14)	0.0330 (16)	0.0119 (14)
C4	0.0472 (19)	0.0245 (14)	0.0442 (18)	0.0081 (14)	0.0263 (15)	0.0093 (13)
C5	0.0335 (17)	0.0273 (14)	0.0319 (15)	0.0063 (12)	0.0156 (13)	0.0069 (11)
C6	0.0330 (16)	0.0345 (15)	0.0304 (14)	0.0003 (13)	0.0198 (12)	0.0007 (13)
C7	0.0423 (19)	0.0214 (14)	0.0312 (16)	−0.0089 (14)	0.0180 (14)	0.0014 (12)
C8	0.053 (2)	0.0215 (13)	0.0309 (15)	−0.0099 (13)	0.0231 (15)	−0.0060 (11)
C9	0.0281 (17)	0.0274 (14)	0.0380 (16)	0.0020 (12)	0.0187 (13)	0.0087 (13)
C10	0.0212 (16)	0.0343 (15)	0.0291 (15)	0.0023 (13)	0.0105 (12)	0.0035 (12)
C11	0.0257 (16)	0.0216 (13)	0.0184 (12)	−0.0003 (11)	0.0062 (11)	−0.0009 (10)
C12	0.0315 (16)	0.0198 (13)	0.0280 (14)	−0.0024 (12)	0.0160 (12)	−0.0011 (11)
C13	0.0257 (15)	0.0178 (12)	0.0309 (14)	0.0012 (11)	0.0147 (12)	0.0008 (11)
C14	0.0320 (17)	0.0243 (13)	0.0353 (16)	0.0066 (12)	0.0158 (13)	0.0033 (12)
C15	0.039 (2)	0.0549 (19)	0.048 (2)	−0.0006 (16)	0.0106 (16)	−0.0014 (16)

### Geometric parameters (Å, °)

**Table:**

Cu1—N1	1.9727 (19)	N10—C10	1.140 (3)
Cu1—N1^i^	1.9727 (19)	C1—C2	1.433 (3)
Cu1—N2^i^	2.0397 (18)	C3—C4	1.349 (3)
Cu1—N2	2.0397 (18)	C3—H3A	0.9500
Cu1—N7	2.821 (2)	C4—H4A	0.9500
O1—C11	1.352 (3)	C5—C6	1.349 (3)
O1—C14	1.460 (3)	C5—H5	0.9500
N1—C1	1.324 (3)	C6—H6	0.9500
N1—C3	1.367 (3)	C7—C12	1.419 (3)
N2—C2	1.333 (3)	C8—C12	1.428 (4)
N2—C5	1.374 (3)	C9—C13	1.421 (3)
N3—C2	1.337 (3)	C10—C13	1.425 (3)
N3—C6	1.372 (3)	C11—C12	1.389 (3)
N3—H3	0.8800	C11—C13	1.395 (3)
N4—C1	1.343 (3)	C14—C15	1.477 (3)
N4—C4	1.364 (3)	C14—H14A	0.9900
N4—H4	0.8800	C14—H14B	0.9900
N7—C7	1.143 (3)	C15—H15A	0.9800
N8—C8	1.143 (3)	C15—H15B	0.9800
N9—C9	1.144 (3)	C15—H15C	0.9800
			
N1—Cu1—N1^i^	180.00	C3—C4—H4A	126.7
N1—Cu1—N2^i^	97.96 (8)	N4—C4—H4A	126.7
N1^i^—Cu1—N2^i^	82.04 (8)	C6—C5—N2	110.0 (2)
N1—Cu1—N2	82.04 (8)	C6—C5—H5	125.0
N1^i^—Cu1—N2	97.96 (8)	N2—C5—H5	125.0
N2^i^—Cu1—N2	180.0	C5—C6—N3	106.1 (2)
N1—Cu1—N7	95.79 (7)	C5—C6—H6	126.9
N1^i^—Cu1—N7	84.21 (7)	N3—C6—H6	126.9
N2^i^—Cu1—N7	88.69 (7)	N7—C7—C12	177.8 (3)
N2—Cu1—N7	91.31 (7)	N8—C8—C12	179.4 (3)
C11—O1—C14	119.25 (18)	N9—C9—C13	179.1 (3)
C1—N1—C3	105.9 (2)	N10—C10—C13	178.6 (3)
C1—N1—Cu1	113.35 (16)	O1—C11—C12	113.8 (2)
C3—N1—Cu1	140.66 (17)	O1—C11—C13	119.61 (19)
C2—N2—C5	105.23 (19)	C12—C11—C13	126.5 (2)
C2—N2—Cu1	110.87 (15)	C11—C12—C7	124.4 (2)
C5—N2—Cu1	143.89 (17)	C11—C12—C8	119.1 (2)
C2—N3—C6	107.6 (2)	C7—C12—C8	116.3 (2)
C2—N3—H3	126.2	C11—C13—C9	121.6 (2)
C6—N3—H3	126.2	C11—C13—C10	121.2 (2)
C1—N4—C4	107.3 (2)	C9—C13—C10	117.2 (2)
C1—N4—H4	126.3	O1—C14—C15	110.4 (2)
C4—N4—H4	126.3	O1—C14—H14A	109.6
C7—N7—Cu1	103.7 (2)	C15—C14—H14A	109.6
N1—C1—N4	110.7 (2)	O1—C14—H14B	109.6
N1—C1—C2	116.9 (2)	C15—C14—H14B	109.6
N4—C1—C2	132.4 (2)	H14A—C14—H14B	108.1
N2—C2—N3	111.0 (2)	C14—C15—H15A	109.5
N2—C2—C1	116.8 (2)	C14—C15—H15B	109.5
N3—C2—C1	132.2 (2)	H15A—C15—H15B	109.5
C4—C3—N1	109.5 (2)	C14—C15—H15C	109.5
C4—C3—H3A	125.2	H15A—C15—H15C	109.5
N1—C3—H3A	125.2	H15B—C15—H15C	109.5
C3—C4—N4	106.6 (2)		
			
N2^i^—Cu1—N1—C1	178.54 (16)	C6—N3—C2—N2	1.3 (3)
N2—Cu1—N1—C1	−1.46 (16)	C6—N3—C2—C1	−179.2 (2)
N7—Cu1—N1—C1	−91.98 (17)	N1—C1—C2—N2	−0.4 (3)
N2^i^—Cu1—N1—C3	2.7 (3)	N4—C1—C2—N2	178.3 (2)
N2—Cu1—N1—C3	−177.3 (3)	N1—C1—C2—N3	−179.9 (2)
N7—Cu1—N1—C3	92.2 (3)	N4—C1—C2—N3	−1.2 (4)
N1^i^—Cu1—N2—C2	−178.76 (15)	C1—N1—C3—C4	0.2 (3)
N7—Cu1—N2—C2	96.90 (16)	Cu1—N1—C3—C4	176.3 (2)
N1—Cu1—N2—C5	−178.8 (3)	N1—C3—C4—N4	−0.1 (3)
N1^i^—Cu1—N2—C5	1.2 (3)	C1—N4—C4—C3	−0.1 (3)
N7—Cu1—N2—C5	−83.1 (3)	C2—N2—C5—C6	0.7 (3)
N1—Cu1—N7—C7	24.02 (19)	Cu1—N2—C5—C6	−179.3 (2)
N1^i^—Cu1—N7—C7	−155.98 (19)	N2—C5—C6—N3	0.1 (3)
N2^i^—Cu1—N7—C7	121.89 (19)	C2—N3—C6—C5	−0.9 (3)
N2—Cu1—N7—C7	−58.11 (19)	C14—O1—C11—C12	−129.2 (2)
C3—N1—C1—N4	−0.2 (3)	C14—O1—C11—C13	54.0 (3)
Cu1—N1—C1—N4	−177.52 (15)	O1—C11—C12—C7	−164.3 (2)
C3—N1—C1—C2	178.7 (2)	C13—C11—C12—C7	12.3 (4)
Cu1—N1—C1—C2	1.4 (3)	O1—C11—C12—C8	10.1 (3)
C4—N4—C1—N1	0.2 (3)	C13—C11—C12—C8	−173.3 (2)
C4—N4—C1—C2	−178.6 (2)	O1—C11—C13—C9	−166.3 (2)
C5—N2—C2—N3	−1.2 (3)	C12—C11—C13—C9	17.2 (4)
Cu1—N2—C2—N3	178.75 (15)	O1—C11—C13—C10	14.4 (4)
C5—N2—C2—C1	179.2 (2)	C12—C11—C13—C10	−162.0 (2)
Cu1—N2—C2—C1	−0.8 (2)	C11—O1—C14—C15	81.6 (3)

Symmetry codes: (i) −*x*+1, −*y*+1, −*z*+1.

### Hydrogen-bond geometry (Å, °)

**Table:**

*D*—H···*A*	*D*—H	H···*A*	*D*···*A*	*D*—H···*A*
N3—H3···N9^ii^	0.88	2.22	3.011 (3)	149
N4—H4···N9^ii^	0.88	2.17	2.967 (3)	150
C3—H3A···N8^iii^	0.95	2.42	3.187 (3)	138

Symmetry codes: (ii) *x*−1, *y*, *z*; (iii) *x*, *y*, *z*−1.
